# The Influence of Climate on Dental Caries

**Published:** 1910-11-15

**Authors:** A. S. Underwood

**Affiliations:** Professor of Dental Surgery, King’s College


### THE INFLUENCE OF CLIMATE ON DENTAL CARIES

BY A. S. UNDERWOOD.
PROFESSOR OF DENTAL SURGERY, KING'S COLLEGE.

The first step towards the prevention of dental caries must be the identification of the conditions that favor the disease. These conditions must necessarily be of a two-fold character: (1) Anything which tends to weaken the natural antibacterial defence of the tooth itself, (2) anything which tends to increase the power and activity of the invading bacterial army.

It appeared to me some time ago that a careful examination of an extended series of skulls of various races and various epochs might throw some light upon this inquiry. The immunity of certain races and the liability of others taken in conjunction with what is known of their diet, surroundings, and mode of life, might result in useful suggestions.

About two years ago I commenced a series of investigations (making careful notes) of the skulls in accessible collections, such as the Royal College of Surgeons and the Natural History Museum, and, without burdening you with numbers of figures, all of which will be published later, I propose to give a summary of my results so far, to this meeting. I started with strong expectations of finding certain definite results, and, as is so often the case, I did not find in the least what I expected.

In the hot belt of the earth, including India, Africa, and Southern China bathing and washing are natural habits because of the heat, and rinsing the mouth after meals, and the use of sticks, tooth-powder, ashes, and salt for cleansing the mouth is almost universal among the natives; while the food is largely rice, and no alcohol is used. In all of them caries is so rare that to all intents and purposes the natives may be regarded as immune. In the Arctic regions the Esquimaux never wash or clean their mouths (Peary), eat seals, walrus, whale, reindeer, fish and seafowl, and drink alcohol. A very large series of Esquimaux skulls show the practical immunity among the natives. In the temperate regions, among the Australian natives, there is the same immunity.

Clearly cleanliness or uncleanliness by itself is not a sufficient explanation, and climate *per se* has little or nothing to do with the question. Immune teeth among natives are always worn down, and the rare cases of caries are always in cheek teeth.

As far as skulls throw a light on the question, I have no doubt that what is called civilization is attended by a rapid increase of caries.

I used to think that the talked-of-increase in the prevalence of caries of late years really meant that nowadays it was more widely detected and reported, but this illusion is now dispelled. A series of English, Scottish, and Welsh skulls of 100 to 150 years old which I examined, I found, though not immune, fairly free from caries. Most of these were skulls of poor people, *post-mortem* and dissecting room subjects, with the exception of a few wealthy murderers.

Among ancient civilizations, I found an appreciable amount of carries among acient Egyptians. Of ancient skulls I have not finished my inquiry, and therefore have not seen so many specimens of Greek and Roman skulls, but caries was certainly not unknown among them.

In modern times people vary considerably in their liability. M. Magitot published in 1867 some very interesting investigations. He concluded that Caucasian races were specially liable; that mixed races were more liable than pure; that transplanted races were specially liable—I do not agree; that aborigines of America, Mexico, Peru and Patagonia were immune; also that Australia, Madagascar, New Caledonia, Malays, and Javanese Island folk were specially immune.

A map of France, 1867, drawn up so as to show the relative proportions in various areas of persons exempted from military service on account of caries shows in Finisterre 60 exempts out of 547,000 conscripts, and in Europe 5,014 exempts out of 450,000 men of like age. Normandy and the provinces of Garonne, Loire, Dordogne, and Seine are the worst districts.

No single cause explains this fact. Food has little to do with it. In Normandy (the worst district) and in Brittany (the best) the inhabitants eat and drink practically the same things.

Rivers have also no effect. The Seine and Garonne districts are very bad, while the Rhone district is comparatively immune. Nor can seaside, elevation, climate, food, or wealth be shown to have much to do with it when considered apart.

As for racial distribution, the Celts, small dark folk have good teeth, while the Kimric races, large blonde folk, have bad teeth.

Poverty and wealth seem no explanation in Great Britain to-day. Town-reared and country-bred people are all equally liable (Welsh mountains and London streets).

### CONCLUSIONS.

The mischief, as far as weakening the tooth defences is concerned, is to a great extent done before the diet of the individual has passed beyond the milk stage.

Although horse, cow, and goat milk contain much more lime than human milk, and although many artificial foods are ideal in the desirability of their constituents, there is no doubt that they are not assimilated nearly so well as mother's milk; it is not what goes into the baby's mouth but what goes into its blood what counts.

Maternal neglect, which is a product of high civilization, is an important factor. Even where the child is breast-fed, Sutherland states that worry and anxiety render the milk unsuitable; and worry and anxiety, the strenuous life, high pressure, luxury, as much as want and distress, are evils attended upon civilization.

Then there is the wholesale preservation of the physically unfit, which is also a practice pertaining to high civilizations.

My general conclusions are that the prevention of dental caries can best be attained by: (1) A return as far as possible to the simpler life; (2) a return as far as possible to maternal breast-feeding; (3) a universal habit from early childhood of cleansing and rinsing the mouth after every meal, and especially at bedtime.

### DISCUSSION.

Mr. F. J. Bennett (London) thought the simple diet was of importance, as it gave the oral secretions a simpler task in acting on the food.

Mr. W. A. Maggs (London) agreed that the causation of dental decay was a complex problem, and doubted if diet alone would prevent it. Persons taking every care in cleansing their teeth and in chewing their food suffered from caries whilst others, entirely disregarding these matters, escaped. Immunity was, in his opinion, largely due to the secretions of the mouth being normal, and free in amount. It was not always correct to say that attrition of the teeth was due to coarse food. The wearing down of the teeth in uncivilized and civilized races was frequently found to be due to an edge-to-edge "bite"—a type of dentition associated with primitive man, and when seen nowadays indicative of sound teeth and virility. Dr. Sim Wallace advocated eating an apple at the end of a meal for the purpose of removing particles of food from the interstices of the teeth. Such a practice was also beneficial in acting as a gentle laxative, and an apple taken daily after breakfast promoted intestinal peristalsis. In the western counties—Somerset, Dorset, and Devon—dental decay was very prevalent amongst the rural population, and was due to drinking the common beverage—cider.

Dr. Wheatley (M. O. H. Shropshire) thought the question of the importance of hardness of water was one that could be easily cleared up. Certainly in the rural districts of Shropshire, where the water was mostly very hard, the teeth were bad. He had heard no evidence whatever that soft water was a cause of dental caries.

Prof. G. Elliot Smith said that during the last ten years his assistants (Drs. Wood Jones and Derry) and himself examined and made notes of more than 15,000 ancient Egyptian and Nubian crania, representing every historical (and prehistoric) period, and had studied the evidences of dental disease. In predynastic times (before 3,400 B. C.), in early dynastic times (until 2,700 B. C.) in Egypt, and until the time of the eighteenth dynasty (*circa* 1,500) in Nubia, dental caries was so extremely rare as to be practically non-existent; yet alveolar abscesses often occurred, because the large quantities of sand contained in the bread and other foods caused early and extreme wearing down of the teeth, and the infection of the exposed pulp cavities with septic material led to alveolar abscess formation. At the time of the Pyramid builders of Lower Egypt (*circa* 2,700 B. C.) dental caries and tartar formation became suddenly so common, that amongst the remains of 500 (mostly aristocratic) people of this date that we examined, only about fifty were free from caries, and the jaws of most of them were riddled with abscess cavities. Amongst the well-to-do Egyptians caries had been common ever since then; and in later times it gradually spread to the lower classes.

Dr.C. Muthu said that behind all causes, such as errors in diet, artificial feeding of infants, etc., the conditions brought in by the present civilization was the chief cause of disease of teeth. So long as the Indians lived their simple primitive life their teeth were excellent. But as soon as they came into contact with European civilization and adopted Western modes of living their teeth went wrong. Primitive life meant not only eating simple foods, but bathing every morning, washing the teeth before and after every meal, morning worship which brought calm and peace of mind and body, early retirement to bed and early rising—in fact, it meant living near the heart of nature. The civilized man had lost the peace of mind. The mind influenced the body. If health meant harmony of various elements of man, disease was caused by disturbance in the relation between body, mind and spirit— including the disease of teeth. So that behind bad water, bad diet and behind those shallow causes lay the root of a new environment brought on by the modern conditions of Western civilization.

Dr. Cyril Howkins (Birmingham) stated that from his personal observations on the semi-civilized natives of South Africa, they had exceedingly good teeth. He was unable to understand why the Jews had such excellent teeth as a rule, unless it was from the fact that they rarely married anyone save of their own race. He had often wondered if one result of the marriage of individuals from two different races was that the teeth of the offspring were inferior to those of the parents, even if the fact that irregularities of the position which commonly occurred from these marriages was put on one side.—*British Medical Journal.*

